# Mirror replication of sexual facial expressions increases the success of sexual contacts in bonobos

**DOI:** 10.1038/s41598-020-75790-3

**Published:** 2020-11-04

**Authors:** Elisabetta Palagi, Marta Bertini, Giulia Annicchiarico, Giada Cordoni

**Affiliations:** 1 Unit of Ethology, Department of Biology, University of Pisa, Via A. Volta 6, 56126 Pisa, Italy; 2 Natural History Museum, University of Pisa, Via Roma 79, 56011 Calci, Pisa, Italy

**Keywords:** Zoology, Animal behaviour

## Abstract

Rapid Facial Mimicry (RFM), one of the possible predictors of emotional contagion, is defined as the rapid, involuntary and automatic replication of a facial expression. Up to now, RFM has been demonstrated in nonhuman animals exclusively during play. Since in bonobos, as in humans, socio-sexuality is a powerful tool for assessing/strengthening inter-individual relationships, we investigated RFM in this domain. Bonobos displayed *silent bared-teeth* (*sbt*, the most common facial expression during sexual contacts) more frequently after the *detection* of an *sbt* emitted by the trigger than in the *no-detection* condition. This is the first demonstration of the presence of RFM during sex. The occurrence of RFM was positively affected by the sex of the partners with female homo-sexual contacts being punctuated by a higher presence of RFM. At an immediate level, RFM increased the duration of homo- and hetero-sexual contacts. This finding suggests that RFM can increase individuals’ potential fitness benefits. By prolonging their sexual contacts, females can strengthen their social relationships thus increasing the probability to obtain priority over resources (RFM indirect fitness benefits). Via longer copulations, males can increase the probability to make females pregnant (RFM direct fitness benefits). In conclusion, in bonobos the access to the partner’s face during sexual contacts (face-to-face, proximate factor) and the role of socio-sexuality in increasing the individual direct and indirect fitness (ultimate factor) could have favoured the evolution of specific sexual facial expressions and their rapid mirror replication. Our findings on bonobos expand the role of RFM well beyond the animal play domain thus opening new scenarios for future comparative studies exploring the evolution of socio-sexuality in humans.

## Introduction

Group living offers beneficial prospects for individual survival in both human and non-human animals. Nevertheless, the social (and physical) environment is relatively unpredictable over time and competition instead of cooperation can arise between group members^1^. Hence, the capacity to quickly perceive emotional states of others and anticipate their actions becomes crucial and permits individuals to synchronize their behaviour^2^. One of the pillars of the behavioural synchronization is Rapid Mimicry (RM), an automatic replication of others’ facial expressions, vocalizations, and body postures^3–6^. From an operational point of view, RM is quantifiable by measuring the recruitment of muscles whose activation determines a specific motor reaction. The same muscle involvement produces the same outcome between interacting individuals. Since the muscle response is extremely rapid (< 1 s), RM is mainly outside the conscious awareness and voluntary control and, therefore, can be considered as a reflex action^3,7^. Although most of the studies on RM focussed on the involvement of muscle movements (“motor mimicry”), some researches have demonstrated that, in humans, a synchronization in heart-rate, pupil-diameter, breathing rhythm and hormonal levels can also occur (“autonomic mimicry”^6,8–10^). From a neurological point of view, many studies underlined that both perception and replication of a motor stimulus involve the Mirror Neuron System (MNS). The Perception–Action Model, theorized by Preston and de Waal^11^, predicts an involvement of MNS that, during the perception of an action or a facial expression, activates shared representations. Briefly, through the involuntary replication of an observed behaviour, the emotional state related to that behaviour may be arisen in the observer while interacting with the trigger. Hence, although the topic is under debate^12^, in some cases, RM is considered a predictor of emotional contagion^13–15^. RM increases with familiarity between the interacting subjects (e.g., playing) and this explains why the individual representation of others’ emotions is also modulated by their social experience^13,16^. For example, during their social interactions, people are likely to mimic more frequently the facial expressions of in-group- than out-group members^6,17^. During the playful interactions of domestic dogs, the distribution of RM was greatest in response to friends, then acquaintances, and lastly strangers^18^. RM between in-group members represents an important tool, because it helps individuals to appropriately interpret the emotional states of others with whom they share resources and exchange social contacts^19^. At a finer scale, the frequency of RM can also be linked to the relationship quality of the interacting subjects^20^; for example, in humans, friends mimic each other’s smiles more than non-friends^21^. RM promotes the development of tighter social relationships that induce individuals to continue mimicking each other thus generating a positive feedback^5,22^. Even in those species showing a solitarily lifestyle, RM can arise when subjects grow up in the same social environment thus having the opportunity to establish social bonds and high levels of familiarity with group companions (orang-utans^23^; sun bears^24^). RM can be also present between subjects belonging to different species that share the same environment and a certain degree of familiarity^25^.

In humans, Rapid Facial Mimicry (RFM, the automatic and rapid replication of others’ facial expressions; < 1 s) has been demonstrated for the expression of happiness and sadness^20^; in nonhuman animals, RFM has been reported in the play domain and, specifically, for the *play face* expression^5^.

Nothing is known about the presence of RFM during sexual contacts in human and nonhuman animals. Yet, there is no other context that has more direct impact on fitness than sex and if we want to understand the evolution of RFM, sex is perhaps the most important context. Certainly, the study of facial expressions emitted by humans during sex is not easy for evident ethical and conventional reasons. However, some studies have shown that during sex humans display particular facial expressions (e.g., frowns, scowls or grimaces) that strongly resemble those emitted during painful situations^26–30^. The question about the human production of sexual facial expressions is whether they can be conceptualized as an expression of positive emotions (enjoyment or pleasure; communicative domain) or, instead, they are a simply ballistic response similar to a reflex-like reaction linked to the orgasm acme (physiological domain)^27^. Because it is difficult to disentangle the communicative and physiological domains, perhaps a proper question combining these two apparently contrasting domains is: can we consider sexual facial expressions as a means that evolution moulded to *express* one’s own affective state (e.g., arousal) to others?

Socio-sexuality plays an integral role in human relationship durability and satisfaction; indeed, sex has been found to be a significant positive predictor of general wellbeing and social relationship quality, independently from cultural factors^28^. In the society of bonobos (*Pan paniscus*), one of the closest human relatives^31^, socio-sexual behaviour plays an important role as well^32^. In this species, sex functions as social glue by repairing^33^ and strengthening inter-individual relationships^34,35^ and by forming preferential bonds between subjects^35^. The social function of sex is supported by the presence of homo-sexual interactions^36^. Although sexual interactions involve all sex combinations, they are particularly frequent between females^37–39^, who represent the core of the social group and are generally dominant over males^35,40^. Through homo-sexual contacts, females form alliances^40^ and increase their levels of cooperation with a wider range of social partners^41^. Bonobos are the only non-human primate species engaging not only in dorso-ventral but also in ventro-ventral genital contacts independently from the sex of the subjects involved^42^. During both types of sexual contacts, face-to-face interactions and specific facial expressions such as *silent bared-teeth* can occur. The *silent bared-teeth* display can be present also during contexts such as fear, nervousness, or hesitation (motivational conflict)^43^. de Waal^44^ hypothesised that the *silent bared-teeth* during sex can convey a message of arousal/pleasure states in bonobos. According to the author, this hypothesis explained why the *silent bared-teeth* did not appear in the initial phase of the sexual interaction, as it would be expected if fear, nervousness or motivational conflict were at the basis of the facial expression. Indeed, fear, nervousness or motivational conflict usually characterize the period immediately preceding a social interaction, before the receiver demonstrates his/her willingness to accept such interaction.

The importance of face-to-face interaction in bonobos has been demonstrated by a recent experiment of Kano and colleagues^45^. By proposing to animals some pictures showing conspecific and allospecific faces (i.e. chimpanzees), the authors demonstrated that bonobos responded to the stimulus in a well-automatic manner by fixing faces/eyes for longer periods and more rapidly compared to chimpanzees. Moreover, Kret et al.^46^, in a study evaluating the attentional bias towards conspecifics’ emotions, found that bonobos were particularly attracted by images conveying positive emotional valence such as pictures showing conspecifics involved in grooming and sexual behaviour.

Due to the difficulty to study this topic in humans and given that bonobos (*Pan paniscus*) are the only nonhuman species frequently engaging in ventro-ventral sexual interactions, addressing RFM in bonobo sexual context can have significant implications for the role of this phenomenon in the evolution of human socio-sexuality.

If the sexual *silent-bared teeth* (*sbt*) is a reflex-like reaction (possibly linked to the orgasm acme) expressing positive emotions which are shared by the partners through an emotional contagion phenomenon, we expect that *sbt* can also rapidly, automatically resonate in the partner’s face (RFM) (*Prediction 1*). Moreover, if RFM is a socially modulated phenomenon, we expect it be affected by the relationship quality (e.g., grooming and female homo-sexual contacts) shared by the interacting subjects: the stronger the relationship, the more frequent the RFM (*Prediction 2*).

Finally, if RFM is indicative of a communicative exchange of sexual rewarding between partners, we expect that RFM can positively affect the duration of the sexual interaction in bonobos (*Prediction 3*).

## Results

### Sexual interactions and facial expressions

**Figure 1:**
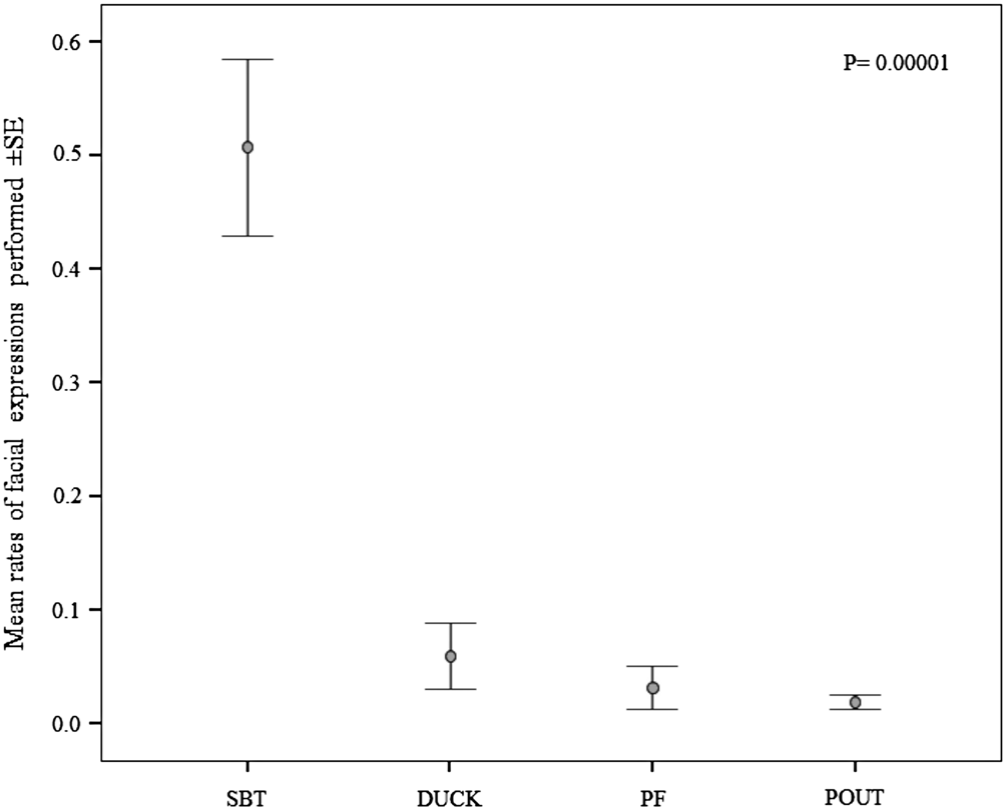
Mean (± SE) of the number of each facial expression performed per sexual contact. Silent Bared-Teeth (SBT), Pout-face (POUT), Duck-face (DUCK), Play Face and Full Play face (PF).

In the bonobos under study, the mean duration of the sexual contacts (*types*: ventro-ventral genito-genital rubbing, ventro-ventral mounting/copulation, dorso-ventral mounting/copulation) was 13.30 secs ± 0.40 SE. The facial expressions performed during sexual contacts were silent bared-teeth (*sbt*), pout-face (*pout*), duck-face (*duck*) and play face (i.e. play face and full play face, *pf*). In order to evaluate which facial display was more frequently performed, we compared the rates of each expression (number of each facial expression on the number of sexual contacts performed) and we found that *sbt* (mean_*sbt*_ 0.51 ± 0.08 SE) was significantly more frequent than the other facial expressions (mean_*duck*_ 0.06 ± 0.03 SE; mean_*pf*_ 0.03 ± 0.02 SE; mean_*pout*_ 0.02 ± 0.01 SE) (Friedman test χ^2^ = 19.206, N = 10, df = 3, p = 0.00001). The post-hoc test revealed that *sbt* was significantly more frequent that any other facial expression recorded during sexual contacts (Bonferroni–Dunnett test: *sbt* > *pout*, q = 2.250, p = 0.001; *sbt* > *pf*, q = 1.950, p = 0.004; *sbt* > *duck,* q = 1.800, p = 0.011; Fig. 1).

### Testing the occurrence of Rapid Facial Mimicry (RFM)

**Figure 2:**
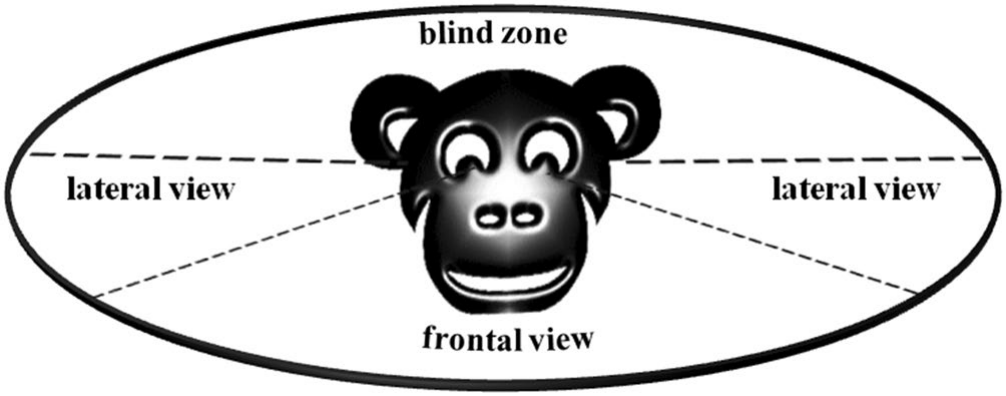
Scheme illustrating the criterion used to evaluate the attentional state of the observer in relation to the detection of *sexual bared-teeth*. Only when the observer was in front of the trigger (direct visual condition) we considered the stimulus as *detected*. All cases of the lateral views were discarded from the analysis.

**Figure 3:**
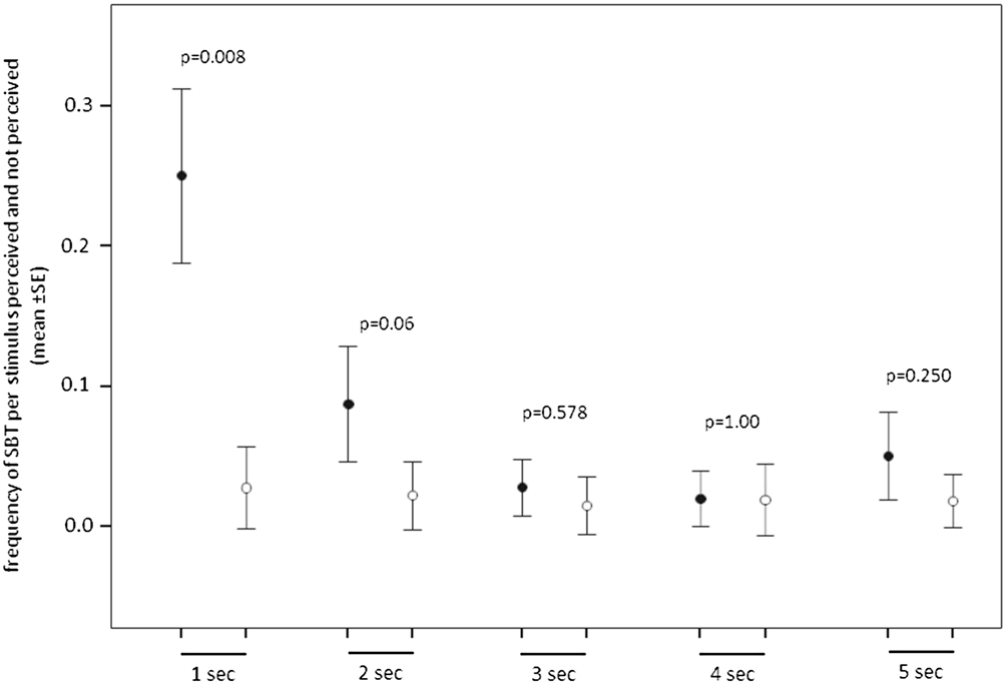
Mean rates (± SE) of *silent bared-teeth* (SBT) performed per stimulus perceived (*detection condition,* direct visual contact between the trigger and the observer, *black dots*) and not perceived (*no-detection condition*, no direct visual contact between the trigger and the observer, open dots) within the 5-s time window.

The aim of this analysis is to verify if the detection of the facial expression *sbt* is able to evoke a mirror response in the receiver (RFM). We found that the rates of *sbt* performed in *detection condition* (direct visual contact between the trigger and the observer, frontal view in Fig. 2) were significantly higher than the rates of *sbt* performed in *no-detection condition* (no direct visual contact between the trigger and the observer, blind zone in Fig. 2) only in the first second after the detection of the stimulus (Randomization paired sample t-test, Bonferroni correction p = 0.01; N_receivers_ = 9; t_1sec_ = − 5.446, p_1sec_ = 0.003; t_2sec_ = − 2.472, p_2sec_ = 0.043; t_3sec_ = − 0.754, p_3sec_ = 0.464; t_4sec_ = − 0.058, p_4sec_ = 1.00; t_5sec_ = − 1.547, p_5sec_ = 0.186; Fig. 3). Only subjects with at least 5 detected and no-detected stimuli were included in this analysis. These results confirm the presence of RFM (defined as a rapid, automatic replication of others’ facial expressions) during bonobo sexual interactions within 1 s after the detection of *sbt* performed by the trigger. The mean latency time of the response was 42.0 csec ± 4.0 SE. Only the *sbt* events occurring within 1 s from the triggering *sbt* stimulus were included in the following analyses.

### Variables affecting the occurrence of RFM

**Figure 4:**
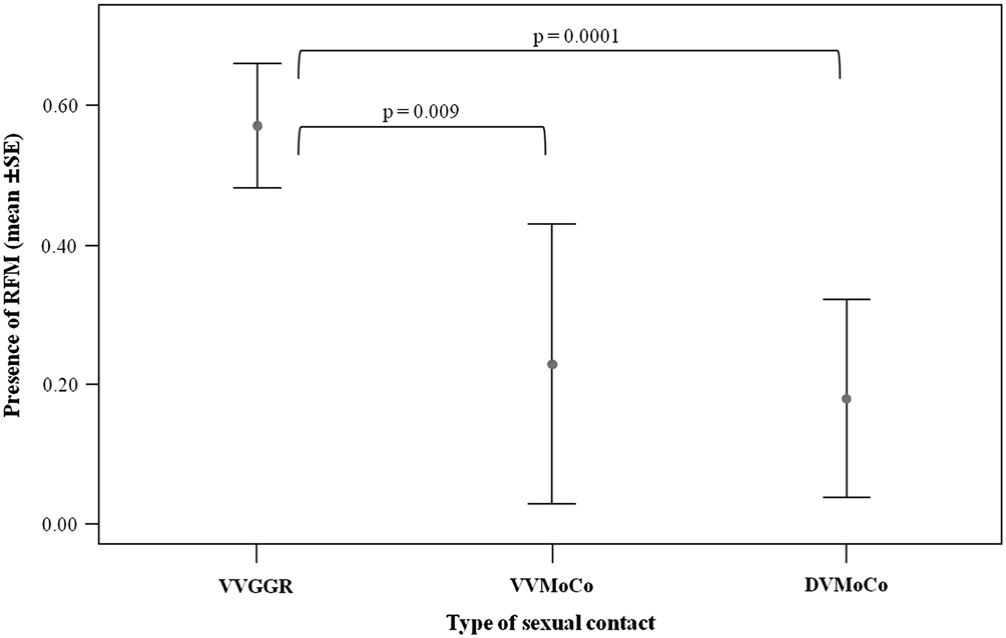
Mean duration (± SE, in seconds) of the sexual contacts as a function of the different types of sexual positions. Ventro-Ventral Genito-Genital Rubbing (VVGGR), Ventro-Ventral Mounting Copulation (VVMoCo), Dorso-Ventral Mounting Copulation (DVMoCo).

The model built to analyze the variables (for the definition see Table 1 and the Method section) possibly affecting the absence/presence of RFM (*absence* = *sbt* perceived but not mimicked by the observer; *presence* = *sbt* perceived and mimicked by the observer within 1 s) significantly differed from the null model (χ^2^ = 21.222, df = 9, p = 0.002). We found that the only factor with a significant effect on the occurrence of RFM was the type of sexual contact (Table 2). The pairwise comparisons via Randomization two independent test revealed that RFM occurred more frequently during ventro-ventral genito-genital rubbing (VVGGR) compared to the other two types of sexual contacts, ventro-ventral mounting copulations (VVMoCo) and dorso-ventral mounting copulations (DVMoCo) (VVGGR vs. VVMoCo t = 2.777, p = 0.009; VVGGR *vs* DVMoCo t = 3.977, p = 0.0001; VVMoCo vs. DVMoCo t = − 0.414, p = 0.713; Fig. 4).

**Table 1:** Description of the variables used in the General Linear Mixed Model (absence/presence of *RFM* as dependent variable). VVGGR = Ventro-Ventral Genito-Genital Rubbing; VVMoCO = Ventro-Ventral Mounting COpulation; DVMoCO = Dorso-Ventral Mounting Copulation.

Name	Type
**Dependent variable**	
Absence/presence of RFM (GLMM)	Nominal (0 = RFM absence – each event of *silent bared teeth* (*sbt)* perceived but not mimicked by the observer; 1 = RFM presence – each event of *sbt* perceived and mimicked by the observer)
**Fixed variables**	
Type of sexual contact	Nominal (VVGGR = 0; VVMoCO = 1; DVMoCO = 2)
Context	Nominal (Prefeeding = 0; Feeding = 1; No-Feeding = 2)
Relationship quality	Scale (dyadic hourly frequency of grooming)
Duration of sexual contact	Scale (secs)
**Random variables**	
DYAD	Nominal
Subgroup	Nominal (RGr_1_ = 1; RGr_2_ = 2; RGr_3_ = 3; LGr_1_ = 4; LGr_2_ = 5; LGr_3_ = 6)

**Table 2:** Influence of the fixed factors on the presence/absence of RFM (dependant variable). VVMoCo = Ventro-Ventral Mounting Copulations; DVMoCo = Dorso-Ventral Mounting Copulations. VVGGR = Ventro-Ventral Genito-Genital Rubbing.

	Estimate	SE	df	Chi-square	P
(Intercept)	− 0.114	0.567	a	− 0.201	0.840
Type of sexual contact (VVMoCo)^b,c^	− 1.506	0.639	2	− 2.357	**0.018**
Type of sexual contact (DVMoCo)^b,c^	− 1.777	0.567	2	− 3.136	**0.002**
Duration of sexual contacts	− 0.015	0.023	1	− 0.671	0.502
Context (feeding)^b,c^	0.528	0.403	2	1.312	0.189
Context (no feeding)^b,c^	0.465	0.601	2	0.774	0.439
Relationship quality	9.492	13.035	1	0.728	0.467

The significant P values are in bold.

^a^Not shown as not having a meaningful interpretation.

^b^Estimate ± SE refer to the difference of the response between the reported level of this categorical predictor and the reference category of the same predictor.

^c^These predictors were dummy coded, with the “VVGGR”, “pre-feeding” being the reference categories.

### Variables affecting the duration of sexual contacts

When analyzing the duration of the sexual contacts (LMM), the full model significantly differed from the null model (χ^2^ = 1999.837, df = 13, p = 0.0005). For the definition of the fixed and random variables see Table 3 and the Method section. We found that the only factor with a significant effect on duration of sexual contacts was the variable *SBT_not perceived_perceived_mimicked* (Table 4)*.* The category *not perceived* included the sexual contacts with at least one silent bared teeth (*sbt*) not perceived by the observer, the category *perceived* involved the sexual contacts with at least one *sbt* perceived but not mimicked by the observer, and the category *mimicked* included sexual contacts with at least one *sbt* perceived and mimicked by the observer within 1 s (for details see Methods).

**Table 3:** Description of the variables used in the Linear Mixed Model Analyses (*Duration of each sexual contact* as dependent variable). VVGGR = Ventro-Ventral Genito-Genital Rubbing; VVMoCO = Ventro-Ventral Mounting COpulation; DVMoCO = Dorso-Ventral Mounting Copulation. ∆NDS = absolute values of Delta Normalized David’s Scores.

Name	Type
**Dependent variable**	
Duration of sexual contact (LMM)	Scale (seconds)
**Fixed variables**	
Type of sexual contact	Nominal (VVGGR = 0; VVMoCO = 1; DVMoCO = 2)
Rank distance	Scale (absolute ∆NDS values)
Context	Nominal (Prefeeding = 0; Feeding = 1; No-Feeding = 2)
Relationship quality	Scale (dyadic hourly frequency of grooming)
SBT_not perceived_perceived_mimicked	Nominal (mimicked—sexual contacts with at least one *silent bared teeth (sbt)* perceived and mimicked by the observer = 2; perceived—sexual contacts with at least one *sbt* perceived but not mimicked by the observer = 1; not perceived = sexual contacts with at least one *sbt* not perceived by the observer = 0)
**Random variables**	
DYAD	Nominal
Subgroup	Nominal (RGr_1_ = 1; RGr_2_ = 2; RGr_3_ = 3; LGr_1_ = 4; LGr_2_ = 5; LGr_3_ = 6)

**Table 4:** Influence of the fixed factors on the duration of the sexual contacts (in seconds, dependent variable). VVMoCo = Ventro-Ventral Mounting Copulations; DVMoCo = Dorso-Ventral Mounting Copulations. VVGGR = Ventro-Ventral Genito-Genital Rubbing.

	Estimate	SE	df	Chi-square	P
(Intercept)	12.383	1.455	a	8.512	0.493
Type of sexual contact (VVMoCo)^b,c^	− 0.749	1.991	2	− 0.376	0.707
Type of sexual contact (DVMoCo)^b,c^	− 1.306	1.052	2	− 1.241	0.216
Not perceived_perceived_mimicked (perceived)^b,c^	2.223	0.989	2	2.247	**0.025**
Not perceived_perceived_mimicked (mimicked)^b,c^	4.626	1.057	2	4.376	** < 0.001**
Context (feeding)^b,c^	− 0.758	0.934	2	− 0.812	0.418
Context (no feeding)^b,c^	0.221	1.206	2	0.183	0.855
Relationship quality	16.964	16.812	1	1.009	0.314
Rank distance	− 0.233	1.139	1	− 0.204	0.838

The significant P values are in bold.

^a^Not shown as not having a meaningful interpretation.

^b^Estimate ± SE refer to the difference of the response between the reported level of this categorical predictor and the reference category of the same predictor.

^c^These predictors were dummy coded, with the “VVGGR”, “Not perceived”, “pre-feeding” being the reference categories.

**Figure 5:**
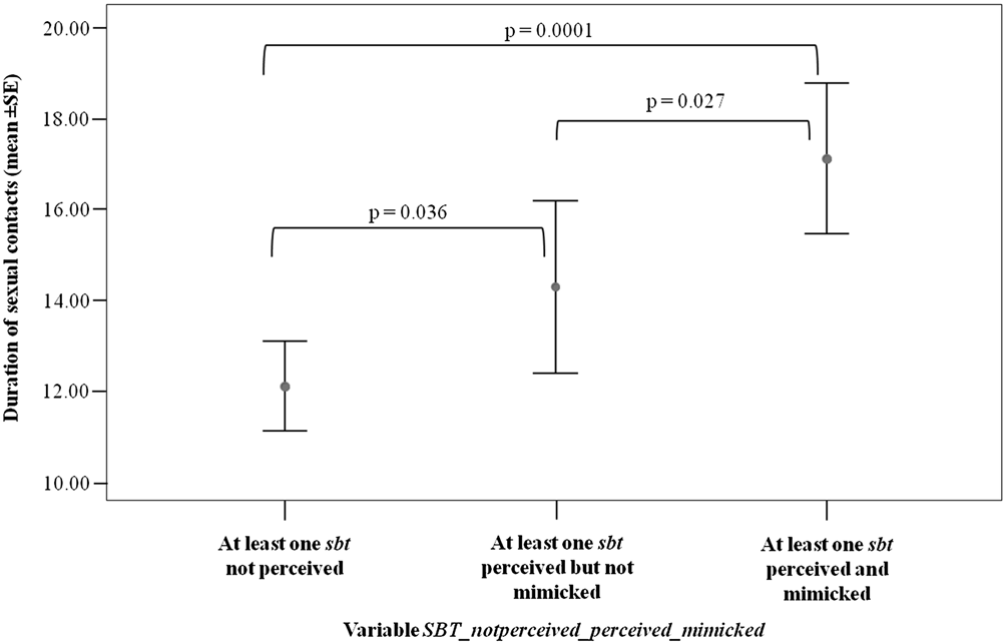
Mean duration (± SE, in seconds) of the sexual contacts in case of i) at least one *silent bared-teeth* (SBT) performed by the trigger but not perceived by the partner; ii) at least one SBT performed by the trigger and perceived by the observer but not mimicked; iii) at least one SBT perceived and mimicked by the observer.

The pairwise comparisons via Randomization two independent test revealed that the duration of the sexual contacts significantly varied across the three categories (perceived > not perceived; t = -2.113; p = 0.036; mimicked > perceived; t = 2.225; p = 0.027; mimicked > not perceived; t = -4.907; p = 0.0001) (Fig. 5).

## Discussion

In bonobos, as well as in humans, socio-sexuality is a powerful tool for assessing and strengthening social bonds between group members^28,35^. The habitual use of sex in the social domain is accompanied by a corresponding complexity in communicative behaviour^47,48^. For example, a recent study has evidenced that people, despite the presence of some cultural variants, produce specific facial expressions of sexual pleasure and arousal^30^. In bonobos, as it occurs in humans^27^, we found that sexual contacts can be punctuated by different facial displays even though the *silent bared-teeth* (*sbt*) is the most representative being performed in more than half of the contacts recorded (Fig. 1). The frequent presence of *sbt* during sexual contacts is in agreement with the data reported by de Waal^44^ for the San Diego bonobo colony. Certainly, independently from the expression performed, the facial expressions of sexual arousal are favoured by face-to-face interactions between partners, a characteristic shared by *Pan paniscus* and *Homo sapiens*^49,50^. Face-to-face engagement may serve to maintain eye contact between partners also when the sexual interaction is performed dorso-ventrally. Indeed, in various monkey species, females frequently look back at males during dorso-ventral copulations actively searching for their gazing^47^. The face-to-face interaction is the prerequisite for the perception of others’ facial expressions and their potential replication. Accordingly, we found that bonobos, after engaging in a face-to-face interaction, can mimic the *sbt* performed by the sexual partner within the first second after the detection of the stimulus (*Prediction 1 supported*; Fig. 3). This is the first demonstration of Rapid Facial Mimicry (RFM) during socio-sexual contacts in primates. This finding supports the hypothesis that *sbt* can have an arousal communicative valence (i.e., enjoyment or pleasure), as this facial expression promptly resonates in the face of the sexual partner. Indeed, the involuntary replication of others’ facial expression can evoke in the observer the same emotional state related to that facial expression^11^.

The relationship quality measured by grooming did not significantly affect the occurrence of RFM in bonobos (*Prediction 2 not supported*). The variable which had a significant effect on the occurrence of RFM was the type of sexual contact. In particular, female homo-sexual ventro-ventral genito-genital rubbing (VVGGR) had a stronger positive effect compared to hetero-sexual ventro-ventral (VVMoCo) and dorso-ventral mounting/copulation (DVMoCo) (Fig. 4). Our finding suggests that it is not the ventro-ventral position which affects the presence of the RFM phenomenon, but rather the involvement of two females in the interaction. Probably, during their VVGGR events, females experience a higher level of emotional state matching (e.g., arousal) compared to hetero-sexual contacts. This is in line with the data on the importance of sex in unrelated bonobo females (female exogamy), who engage in homo-sexual behaviour to form alliances, based on the development and maintenance of high quality social relationships^39,51–54^.

The results from the first model (GLMM) suggest that the occurrence of RFM is not a byproduct of the duration of the sexual contact. However, the presence of the mimicking response of *sbt* prolonged the duration of the sexual interactions compared to the presence of *sbt* not perceived or perceived but not mimicked by the observer (*Prediction 3 supported*; Fig. 5). It is likely that through the automatic replication of *sbt*, the sexual arousal experienced by the trigger can be shared with the partner. In this view, it seems that *sbt* can fairly communicate an emotional engagement necessary to prolong the sexual interaction. A similar immediate effect of RFM on the duration of the interaction has been found in the play domain. In chimpanzees, gorillas^55^, geladas^56,57^, Tonkean macaques^58^, domestic dogs^18^ and meerkats^59^, the rapid and unconscious replication of the play faces significantly prolonged the playful sessions. In bonobos, during sex RFM could play the immediate role of synchronizing/coordinating the motor actions between partners and, thereby, facilitating their reciprocal involvement. The arousal and motor reciprocal engagement can translate into prolonged and successful homo- and hetero-sexual interactions.

This is the first evidence of a connection between RFM and potential direct and indirect fitness benefits. On one hand, through prolonged VVGGR, females can strengthen their social relationships thus increasing the probability to obtain priority over resources (indirect fitness benefits of RFM). On the other hand, via longer copulations, males can increase the probability to make females pregnant (direct fitness benefits of RFM), also considering that prolonged copulations are good indicators of ejaculation in primates^60^.

In conclusion, in bonobos the possibility to have access to the partner’s face during sexual contacts (face-to-face, proximate factor) and the important role of socio-sexuality in increasing the individual direct and indirect fitness (ultimate factor) could have favoured the evolution of a specific sexual facial expression, the *silent bared teeth,* and its automatic mirror replication, one of the processes possibly at the basis of emotional contagion. Our findings on bonobo sexuality expand the role of RFM well beyond the animal play domain thus opening new scenarios for future comparative studies exploring the evolution of socio-sexuality in humans.

## Methods

### Ethic statement

The Wilhelma Zoo (Stuttgart, Germany) gave the permission for the data gathering. The research complied with current laws of Germany, Italy, and the European Community. The University of Pisa (OPBA committee) waived the need for a permit since the study was purely observational with no manipulation of animals whatsoever*.* All the observational methods were carried out in accordance with relevant guidelines and regulations.

### The study group

The bonobo group was hosted at the Wilhelma Zoo (Stuttgart, Germany) and was composed by 16 individuals (Table S1). The colony was separated into two sub-groups labelled as “*Right Group*” (*RGr*) and “*Left Group*” (*LGr*) in relation to the indoor facility they occupied. The composition of both sub-groups changed once per month in order to maintain group fluidity. During the observation period, we followed six different sub-groups (*RGr*_*1*_, *RGr*_*2*_, *RGr*_*3*_, *LGr*_*1*_, *LGr*_*2*_, *LGr*_*3*_; Table S2). Each sub-group occupied an enclosure of about 350 m^2^ and the two sub-groups were in both visual and auditory contact through the glass parts of the dividing wall. Although platforms, ropes, trunk, straw and other environmental enrichments were present in each enclosure, they did not preclude good visibility to the observers. The bonobos were fed three times per day (08:45 a.m., 11:30 a.m., 03:00 p.m.) and received fruit, vegetables and yogurt. The water was available ad libitum.

### Data collection

We collected data from November 2017 to March 2018. The observations took place daily over a 6-h period, spanning morning and afternoon and including feeding times. Before commencing systematic data collection, the two observers (G.A., M.B.) underwent 30 h of training to become skilled in individual and behavioural pattern identification (grooming and sexual contacts). During the training period, the two observers collected data simultaneously and the behavioural patterns were compared. Training ended when the observations produced a Cohen’s K = 0.85^61^.

Via scan animal sampling^62^ we collected grooming every 10 min for all animals in each sub-group. We registered the interacting subjects and the directionality of grooming. This yielded 288 h of observation for *LGr*_*1*_, *LGr*_*2*_, *LGr*_*3*_ and 309 h for *RGr*_*1*_, *RGr*_*2*_, *RGr*_*3*_.

Data on aggression were collected via all occurrences sampling method^62^. For each agonistic event, we recorded the identity of the winner (the subject that never displayed submissive and/or fear patterns) and the loser (the subject that displayed submissive and/or fear patterns). We discarded from the analysis all the conflicts without a clear outcome. All occurrences sampling method was also used to obtain detailed data on socio-sexual behaviours, thus all instances of sexual invitations and contacts occurring during the observation period were recorded. Sexual invitations are different in males and females. Males generally sit and slap the feet on the ground while maintaining their legs opened. They can swing their torsos while showing their erect penis. Females usually walk slowly in front of males while watching back towards them. Then, they can lay down or crouch in front of males^50^. Due to the presence of sexual invitations, sexual contacts in bonobos were easily predicted and the observers could easily anticipate the forthcoming bout. This permitted observers to turn on the camera well before the beginning of the sexual contact. Moreover, when sexual contacts occurred repeatedly and involved different subjects, the camera worked *in continuum* to avoid losing interactions. The good visibility due to the absence of any visual obstacles permitted yielding 944 sexual contacts (from the first to the last contact of the genitals of the two partners) including all the subjects of the colony, including immatures.

The videotaped sequences including only the 10 adults were then analysed and coded using the program VideoLAN Client 2.2.1 and Jump-to-Time, which is a VLC extension. Before commencing systematic analysis of the videotaped sequences, the two observers (G.A., M.B.) underwent a second training. The training lasted one month (three times per week) for a total period of 60 h (April 2018). For the following five months (May–September 2018), at the beginning of each week, we checked for the observation reliability for all the facial expressions (*sbt*; *pout*; *duck*; *pl*), sexual contacts (*Ventro-Ventral Genito-Genital Rubbing; Ventro-Ventral Mounting Copulation, Dorso-Ventral Mounting Copulation*) and the *detection/no-detection* conditions (see below for the definitions). The Cohen’s value was never below 0.80 for each of the behavioural patterns and for the two conditions considered for the study.

### Operational definitions and statistics

A sexual interaction began when the genitals of the two partners entered in contact and ended when one of the partners moved away. If the two subjects engaged in a second interaction, this session was considered as new. For each sexual interaction we recorded (a) the identity of the partners, (b) the type of sexual interaction performed (c) the position of subjects (upper position, *up*; lower position, *low*), (d) the facial expressions emitted in the exact chronological order (e) the exact timing of each facial expression with a 2-csec accuracy and, (f) the duration of sexual interaction (seconds).

#### Facial expressions during sexual contacts

For each facial expression performed (*sbt*; *pout*; *duck*; *pf*) we determined the exact duration via frame-by-frame video analysis (accuracy 2 csec) by using the Jump-to-Time Previous frame v3 (addons.videolan.org), which is VLC Extension. The duration of *sbt* and *pf* was calculated from the first frame showing the separation of the inferior from the superior lip until the first frame showing the two lips closed again. The duration of *pout* and *duck* was calculated from the first frame showing the protrusion of the lips until the first frame showing the two lips relaxed. To verify that *sbt* was actually the most represented facial expression during sexual interactions^44^ also in our study group, we divided the number of each facial expression recorded on the total number of sexual interactions performed by individuals. Due to the non-normality of the data (Anderson–Darling, p < 0.05, EasyFit 5.5 Professional), we applied the Friedman test to compared the distribution of facial expressions.

#### Demonstrating the presence of RFM phenomenon

To verify the occurrence of Rapid Facial Mimicry (RFM) during sexual interactions, we distinguished two different conditions: *detection* and *no-detection* (Fig. 2). In the *detection condition,* we recorded the number of *silent bared-teeth* (*sbt*) performed by the subject when the face of the trigger (defined as the first individual who emitted the *sbt* stimulus) was directed towards the face of the receiver (direct visual contact condition, detection of the *sbt* stimulus). The subjects could engage in direct visual contact during both the ventro-ventral and dorso-ventral sexual contacts. In the last case, the subject in the lower position (a female) turned her head to engage in a face-to-face contact with the subject occupying the upper position.

In the *no-detection condition,* we recorded the number of *sbt* performed by the subject when he/she was facing away from the trigger who previously emitted the *sbt* stimulus (without direct visual contact condition, no-detection of the *sbt* stimulus). If the partner, who was initially looking away, turned his/her face towards the trigger that was still emitting an *sbt*, this case was categorized as *detection condition*, because the partner actually perceived the stimulus. All the doubtful cases (n = 163) linked to lateral views were discarded from the analyses (Fig. 2).

Then, we divided the number of facial expressions performed in the *detection* and *no-detection* condition by the number of stimuli detected and not detected, respectively. The rates obtained for each condition were compared via randomization paired samples t-tests. The RFM latencies were measured frame-by-frame starting from the onset of the trigger *sbt* stimulus and ending with the onset of the observer’s *sbt* response both in *detection* and *no-detection* condition (with 2-csec accuracy). The comparison was done sec-by-sec within a 5-s time-window. We employed Bonferroni correction to adjust for multiple comparisons.

### Variables affecting the occurrence of the Rapid Facial Mimicry

To verify which variable affected the occurrence of RFM (presence/absence), we ran a General Linear Mixed Model (GLMM) by using the *lme4* package^63^ in R (R Core Team, 2019; version 3.6.1). In this model, the response variable was binomial (*absence* = each *sbt* event perceived but not mimicked by the observer, N = 91; *presence* = each *sbt* event perceived and mimicked by the observer within 1 s, N = 78). The prerequisite for this analysis was that the stimulus emitted by the trigger (*sbt*) had to be perceived by the partner. Therefore, all *sbt*_s_ performed but not perceived were excluded from this analysis. The dataset used for this analysis was N = 169 (all the *sbt* perceived).

The following variables have been used as fixed factors (Table 1).

#### Type of sexual contact

We categorized the main types of sexual contacts as follows: Ventro-Ventral Genito-Genital Rubbing (VVGGR, female homo-sexual contact; number of *sbt*_*s*_ perceived occurring during VVGGR = 122), Ventro-Ventral Mounting Copulation (VVMoCO, hetero-sexual contact; number of *sbt*_*s*_ perceived occurring during VVMoCo = 18), Dorso-Ventral Mounting Copulation (DVMoCO, hetero-sexual contact; number of *sbt*_*s*_ perceived occurring during DVMoCO = 29)^51^.

#### Rank distance (ΔNDS)

The NDS values were calculated on the basis of a dyadic dominance index (D*ij*) in which the observed proportion of wins (P*ij*) is corrected for the chance occurrence of the observed outcome which is calculated on the basis of a binomial distribution with each subject having an equal chance of winning or losing in every dominance encounter^64^. The correction is necessary when, as in the case of our study group, the interaction numbers greatly differ between dyads. We determined the NDS-based hierarchy by ranking the individuals according to their NDS values. For each dyad, we calculated the absolute value of the ΔNDS.

#### Context

We defined as “Prefeeding” the last 30-min block before the food provisioning and as “Feeding” the 30-min block starting from food provisioning. The parameter for delimiting the two periods linked to feeding activity was the usual time span necessary for complete food consumption^65^. “No-feeding” context included all the activities performed by the animals outside the “Prefeeding” and “Feeding” contexts.

#### Relationship quality

The quality of the relationship between the subjects forming each dyad (A-B) was determined by calculating the ratio between the number of the grooming sessions and the total number of scans in which at least one of the subjects of the dyad was present.

#### Duration of sexual contacts

The duration of each sexual contact was measured in seconds.

To exclude the occurrence of collinearity among the fixed factors, we examined the variance inflation factors (VIF package^66^). Since collinearity has been found between the relationship quality and rank distance variables, this second factor was excluded from the analysis. We confirmed that the GLMM was not overdispersed (chi-sq = 170.298, p = 0.274, dispersion parameter = 1.06).

### Variables affecting the duration of sexual contacts

To verify which variable affected the duration of the sexual contacts, we ran a Linear Mixed Model (LMM) by using the *lme4* package^63^ in R (R Core Team, 2019; version 3.6.1). To analyze the duration of the sexual contacts (dependent variable), we used the dataset including 305 sexual events. For this model, we verified the distribution and homogeneity of the residuals by looking at the qqplot and plotting the residuals against the fitted values (a function written by R. Mundry). The distribution of the duration of each sexual contact was normal. To exclude the occurrence of collinearity among predictors, we examined the variance inflation factors (VIF package;^66^). No collinearity has been found between the fixed factors (Max VIF = 1.19).

The following variables have been used as fixed factors (Table 3): *Type of sexual contact* (N_VVGGR_ = 208; N_VVMoCo_ = 15; N_DVMoCo_ = 82), *Rank distance (ΔNDS), Context*, *Relationship quality,* and *SBT_not perceived_perceived_mimicked)*.

The variables *Type of sexual contact*, *Rank distance (ΔNDS), Context*, *Relationship quality* have been defined above.

#### SBT_not perceived_perceived_mimicked

The category not perceived included the sexual contacts with at least one silent bared teeth (*sbt*), but none of the *sbt*_*s*_ were perceived by the observer (N = 191). The category *perceived* involved the sexual contacts with at least one *sbt* perceived but not mimicked by the observer (N = 59). The category *mimicked* included sexual contacts with at least one *sbt* perceived and mimicked by the observer within 1 s (N = 55) (Table 3). Since each sexual contact could include more than one of these categories, we treated them as mutually exclusive. If a sexual contact included both *sbt* not perceived and perceived but not mimicked, such event fell in the *perceived* category. If a sexual contact included both *sbt* perceived and *sbt* perceived/mimicked, such event fell in the *mimicked* category. This procedure avoided the pseudo-replication of the data.

The random factors included in both GLMM and LMM were each dyad involved in the sexual contact and the subgroups (Tables 1 and 3).

For both models, we tested the significance of the full model^67^ by comparing it against a null model comprising only the random factor, by using a likelihood ratio test (Anova with argument test "Chisq"^68^). Then, we calculated the p-values for the individual predictors based on likelihood ratio tests between the full and the respective null model by using the R-function "drop1"^69^. We performed all pairwise comparisons via the randomization procedures (two-independent sample test; Resampling Procedures 1.3, David C. Howell, Freeware; 10,000 permutations).

## Supplementary information

Supplementary Information 1.

Supplementary Information 2.

Supplementary Information 3.

Supplementary Information 4.

## Data Availability

All data generated or analysed during this study are included in this published article (and its Supplementary Information files).
