# Supplementary material for: Mirror replication of sexual facial expressions increases the success of sexual contacts in bonobos
Source: Sci Rep. 2020 Nov 4;10:18979. doi: 10.1038/s41598-020-75790-3 (PMC7643078; doi:10.1038/s41598-020-75790-3)
Supplement: Supplementary file 1 — Supplementary Information 1. [file 41598_2020_75790_MOESM1_ESM.docx]

| Subject | Kinship | Sex | Class | Date of birth |
| --- | --- | --- | --- | --- |
| Banbo | Yanola mother | F | Adult | 2002 |
| Bobali |  | M | Immature | 2013 |
| Chimba | Koju mother | F | Adult | 1995 |
| Chipita | Kasai mother | F | Adult | 1991 |
| Fimi |  | F | Adult | 2008 |
| Haiba |  | F | Adult | 2001 |
| Huenda | Makasi mother | F | Adult | 2006 |
| Kasai | Chipita son | M | Adult | 2004 |
| Kolela | Liboso daughter | F | Immature | 2016 |
| Kombote |  | F | Adult | 1966 |
| Koju | Chimba son | M | Newborn | 2017 |
| Liboso | Kolela, Lubao mother | F | Adult | 1997 |
| Lubao | Liboso son | M | Immature | 2013 |
| Makasi | Huenda son | M | Immature | 2015 |
| Mobikisi |  | M | Adult | 1980 |
| Yanola | Banbo daughter | F | Immature | 2016 |

**Table S1 –** The colony of bonobos hosted at the Wilhelma Zoo (Stuttgart, Germany)
