# Supplementary material for: Mirror replication of sexual facial expressions increases the success of sexual contacts in bonobos
Source: Sci Rep. 2020 Nov 4;10:18979. doi: 10.1038/s41598-020-75790-3 (PMC7643078; doi:10.1038/s41598-020-75790-3)
Supplement: Supplementary file 2 — Supplementary Information 2. [file 41598_2020_75790_MOESM2_ESM.docx]

| RGr1 | RGr2 | RGr3 | LGr1 | LGr2 | LGr3 |
| --- | --- | --- | --- | --- | --- |
| \| Kasai \| \| --- \| \| Haiba \| \| Liboso \| \| Chipita \| \| Chimba \| \| Kolela \| \| Lubao \| \| Bobali \| | \| Kasai \| \| --- \| \| Haiba \| \| Liboso \| \| Chipita \| \| Kolela \| \| Lubao \| \| Bobali \| \| Huenda \| \| Makasi \| | \| Kasai \| \| --- \| \| Liboso \| \| Kolela \| \| Lubao \| \| Bobali \| \| Chipita \| \| Huenda \| \| Makasi \| \| Chimba \| \| Koju \| | \| Mobikisi \| \| --- \| \| Kombote \| \| Huenda \| \| Banbo \| \| Fimi \| \| Yanola \| \| Makasi \| | \| Mobikisi \| \| --- \| \| Kombote \| \| Banbo \| \| Fimi \| \| Yanola \| \| Chimba \| \| Koju \| | \| Mobikisi \| \| --- \| \| Kombote \| \| Banbo \| \| Yanola \| \| Fimi \| \| Haiba \| |

**Table S2** - Composition of the different bonobo sub-groups during the observation period
